# n-3 fatty acid-enriched parenteral nutrition regimens in elective surgical and ICU patients: a meta-analysis

**DOI:** 10.1186/cc11668

**Published:** 2012-10-04

**Authors:** Lorenzo Pradelli, Konstantin Mayer, Maurizio Muscaritoli, Axel R Heller

**Affiliations:** 1 AdRes HE&OR, Piazza Carlo Emanuele II 19, I-10123, Turin, Italy; 2 Lung Transplant Program, Internal Medicine, Pulmonary Medicine, Intensive Care Medicine, Sleep Medicine. Department of Internal Medicine, Justus-Liebig University Giessen, Klinikstrasse 36, D-35392, Giessen, Germany; 3 Internal Medicine, Università La Sapienza, Via del Policlinico, 155, I-00161, Rome, Italy; 4 Clinic for Anaesthesiology and Intensive Therapy, University Dresden, Fetscherstraße 74, D-01307 Dresden, Germany

## Abstract

**Introduction:**

Previous studies and a meta-analysis in surgical patients indicate that supplementing parenteral nutrition regimens with n-3 polyunsaturated fatty acids (PUFAs), in particular eicosapentaenoic acid (EPA) and docosahexaenoic acid (DHA), is associated with improved laboratory and clinical outcomes in the setting of hyper-inflammatory conditions. Refined or synthetic fish oils are commonly used as a source of EPA and DHA. The objective of the present meta-analysis was to evaluate n-3 PUFA-enriched parenteral nutrition regimens in elective surgical and intensive care unit (ICU) patients.

**Methods:**

Medline was searched for randomized controlled trials comparing n-3 PUFA-enriched lipid emulsions with standard non-enriched lipid emulsions (i.e. soybean oil, MCT/LCT or olive/soybean oil emulsions) in surgical and ICU patients receiving parenteral nutrition. Extracted data were pooled by means of both random and fixed effects models, and subgroup analyses were carried forward to compare findings in ICU versus non-ICU patients.

**Results:**

A total of 23 studies (n = 1502 patients: n = 762 admitted to the ICU) were included. No statistically significant difference in mortality rate was found between patients receiving n-3 PUFA-enriched lipid emulsions and those receiving standard lipid emulsions (RR= 0.89; 0.59, 1.33), possibly reflecting a relatively low underlying mortality risk. However, n-3 PUFA-enriched emulsions are associated with a statistically and clinically significant reduction in the infection rate (RR =0.61; 0.45, 0.84) and the lengths of stay, both in the ICU (-1.92; -3.27, -0.58) and in hospital overall (-3.29; -5.13, -1.45). Other beneficial effects included reduced markers of inflammation, improved lung gas exchange, liver function, antioxidant status and fatty acid composition of plasma phospholipids, and a trend towards less impairment of kidney function.

**Conclusions:**

These results confirm and extend previous findings, indicating that n-3 PUFAs-enriched parenteral nutrition regimens are safe and effective in reducing the infection rate and hospital/ICU stay in surgical and ICU patients.

## Introduction

The role of polyunsaturated fatty acids (PUFA) in the modulation of biologic activities was identified some decades ago, starting from the first studies on the lower cardiovascular risk found in populations with an extremely high intake of n-3 PUFA such as eicosapentaenoic acid (EPA), and docosahexaenoic acid (DHA) [1,2]. In addition to being structural constituents of cell membranes, PUFA are precursors of biological mediators involved in the regulation of many physiological functions, including immune response, blood pressure regulation, cell proliferation, blood clotting, and inflammation [3,4]. The balance between n-3 and n-6 PUFA is important, as mediators derived from the n-6 PUFA (mainly arachidonic acid, AA) favour an inflammatory response, while mediators stemming from n-3 PUFA such as EPA and DHA exert less pro-inflammatory actions.

Intravenous lipid emulsions have been established for many years as an integral part of parenteral nutrition due to their high energy density and low osmolarity. These emulsions are traditionally based on vegetable oils that are rich in n-6 fatty acids, such as soybean oil. In recent years, many trials have explored whether parenteral nutrition regimens supplemented with n-3 PUFA may be beneficial in those clinical conditions that are characterized by an inflammatory over-response, for example, sepsis or pancreatitis, and after major abdominal surgery. Refined or synthetic fish oils are commonly used as a source of EPA and DHA and have been incorporated into a new generation of mixed lipid emulsions.

Chen *et al*. published a meta-analysis of trials conducted in patients undergoing major abdominal surgery and found evidence that parenteral n-3-enriched lipid emulsions in the setting of total parenteral nutrition are beneficial in terms of relevant clinical outcomes, such as infection rate and hospital length of stay (LOS) [5]. However, several studies evaluating the effects of fish oil-based emulsions in clinical conditions in which such hyper-inflammation is a characteristic were omitted from this meta-analysis. Moreover, additional clinical studies have been published in the interim.

The objective of our study was to provide an updated and more extensive analysis of the available evidence on the clinical efficacy and safety of n-3 PUFA-enriched parenteral lipid emulsions in elective surgical and ICU patients, as compared to standard (non-enriched) lipid emulsions, namely, soybean oil, medium-chain triglycerides (MCT)/long-chain triglycerides (LCT) or olive/soybean oil emulsions.

## Materials and methods

The Pubmed database was searched for relevant papers with the following search string: ("Fatty Acids, Omega-3"[Mesh] OR "Fish oil") AND "Parenteral Nutrition"[Mesh] AND ("Surgical Procedures, Operative"[Mesh] OR "Sepsis"[Mesh] OR "Systemic Inflammatory Response Syndrome"[Mesh] OR "Intensive Care Units"[Mesh]). Identified papers were checked for coherence with the defined inclusion criteria, and the reference list of those deemed relevant was manually searched for further relevant studies.

To be included in the present analysis, the retrieved papers were required to report on the results of randomized clinical trials (RCTs) comparing n-3 PUFA-enriched lipid emulsions with standard non-enriched lipid emulsions (that is, soybean oil, MCT/LCT or olive/soybean oil emulsions) in adult ICU patients and/or in elective surgery patients, in terms of clinical outcomes, markers of inflammation and antioxidant status, fatty acid composition of plasma phospholipids, and/or routine laboratory parameters (Table 1).

**Table 1:** Considered outcomes and definitions

Outcome	Definition
Mortality	Number of deaths as reported/patients receiving at least one treatment
Infection rate	Number of nosocomial infections/patients receiving at least one treatment dose
Hospital length of stay (LOS)^a^	Mean (SD) number of hospital days from hospitalization (or intervention) to discharge
ICU LOS^a^	Mean (SD) number of ICU days
Transfused blood units	Standard units
Oxygenation index	Mean (SD) ratio of partial oxygen pressure (PO_2_): inspired oxygen fraction (FiO_2_)
*Serum parameters*	
Alpha-tocopherol	Mean (SD) serum concentration, µmol/L
Aspartate aminotransferase (AST)	Mean (SD) serum concentration, IU/L
Alanine aminotransferase (ALT)	Mean (SD) serum concentration, IU/L
Bilirubin	Mean (SD) serum concentration, mg/dL
C-reactive protein (CRP)	Mean (SD) serum concentration, mg/dL
Creatinine	Mean (SD) serum concentration, mg/dL
Interleukin (IL)-6 change	Mean (SD) difference in serum IL-6 levels between end and beginning of infusion, pg/mL
Lactate	Mean (SD) serum concentration, mmol/L
Triglycerides	Mean (SD) serum concentration, mg/dL
Urea	Mean serum concentration, mmol/L
*Other laboratory parameters*	
Leukotriene B5 (LTB5)	*Ex-vivo *production by leukocytes^b^,
Leukotriene B4 (LTB4)	
LTB5/LTB4 ratio	*Ex-vivo *production by leukocytes
Eicosapentaenoic acid (EPA)	Content in plasma phospholipids, (% of total concentration)
Docosahexaenoic acid (DHA)	
Arachidonic acid (AA)	
Prothrombin time, PT (Quick)	Laboratory standard
Partial thromboplastin time (PTT)	Laboratory standard
Platelets	Count, × 10^3^/µL

^a^When reported as median and interquartile range, data were transformed into mean (SD) by fitting a Weibull distribution. ^b^Analysed in terms of standardised difference as expressed in heterogeneous measurement units.

Identified papers were checked to identify whether results of a single study were published more than once, in order to avoid double imputation. In such instances, the secondary publications were considered only for parameters that were not reported by the main publication. Results were analysed both overall, and by subgroup (non-ICU patients versus ICU patients). Allocation to the ICU subgroup was driven by the explicit mentioning in the published paper of an ICU stay, either in the Methods or in the Results section.

Data were extracted from the text, tables and figures of the original published papers, without any effort to retrieve further data by contacting the authors. Mean and SD were used for the meta-analysis: where these were not reported, they were calculated by fitting an appropriate distribution to the available data. In case of a missing SD only, this value was imputed based on the average SD/mean ratio of included studies reporting on the same parameter. In the case of more than half of the studies not reporting the SD, analysis was not conducted on that parameter. Data pooling was performed with the use of classical meta-analytic methodology, using the RevMan 5.1 software developed for the Cochrane Collaboration. The primary analysis was conducted with random effects models, with Mantel-Haenszel weighting for binary outcomes, and inverse variance weighting for continuous parameters. Exploratory analyses based on fixed effects models were also conducted.

## Results

The original search (August 2011) in PubMed yielded 52 hits. Of these, thirty-seven publications were excluded, as they were not consistent with the inclusion criteria: thirteen were not clinical trials (eleven reviews, one case report, one drug development study), three did not report on any of the analysed outcomes, eight were not randomized, in two the supplementation was administered enterally or orally, in four the study population was not consistent with the specified criteria, and finally, seven studies were not conducted in human subjects. The manual search of the reference lists of the remaining fifteen studies yielded a further eight relevant studies (Additional file 1).

Thus, a total of 23 studies, including a total of 1,502 patients, were included in the meta-analysis: 13 [6-18] of these were conducted in patients admitted to the ICU (n = 762), and 10 [19-28] in patients undergoing major abdominal surgery and not admitted to ICU (n = 740) (Table 2).

**Table 2:** Studies evaluating n-3 PUFA-enriched lipid emulsions for parenteral nutrition and reported outcomes/measured parameters

Study	Setting (n)	n-3 PUFA-enriched lipid emulsion	Standard lipid emulsion	Clinical outcomes	Laboratory outcomes
ICU patients^a ^(n = 762)					
**Antebi 2004 [**6**]**	Major surgery (20)	SO/MCT/OO/FO^b^	SO		AST^c^, ALT^c^, CRP, alpha-T,TG^c^
**Barbosa 2010 [**7**]**	Sepsis (23)	SO/MCT/n-3 TGs^d^	SO/OO	H LOS, ICU LOS, Mortality	CRP, EPA, DHA, AA, LTB4, AST, ALT, Bilirubin, OI, IL-6, PTT, Lac
**Berger 2008 [**8**]**	Abdominal aortic aneurysm (24)	SO/MCT/n-3 TGs^d^	SO/OO	Mortality, H LOS, ICU LOS	EPA, DHA, AA, alpha-T, CRP, TG
**Friesecke 2008 [**9**]**	Critical medical (165)	SO + FO^e^	SO/OO	Mortality, Infection rate, ICU LOS, Bleeding events	IL-6^f ^TBU
**Heller 2004 [**10**]**	Elective colorectal (44)	SO + FO^e^	SO	ICU LOS	AST, ALT, CRP, Bilirubin, PT (Quick), PTT, TBU
**Morlion 1996 [**11**]**	Gastric carcinoma (20)	SO + FO^e^	SO		AA, EPA, DHA, LTB5, LTB4
**Piper 2009** **[**12**]**	Major abdominal or craniomaxillofacial surgery (44)	SO/MCT/OO/FO^b^	SO/OO		AST, ALT, TG
**Roulet 1997 [**13**]**	Elective oesophagectomy (19)	SO + FO^e^	SO		EPA, DHA, AA, BT
**Sabater 2011 [**14**]**	Acute respiratory distress syndrome (44)	SO/MCT/n-3 TGs^d^	SO	Mortality	LTB4
**Wachtler 1997 [**15**]**	Elective abdominal surgery (40)	SO/MCT/n-3 TGs^d^	SO/OO	Infection rate, H LOS, ICU LOS	LTB4, LTB5, LTB ratio, IL-6^f^
**Wang 2008 [**16**]**	Severe acute pancreatitis (40)	SO + FO^e^	SO	Mortality, Infection rate, H LOS, ICU LOS	EPA, CRP, OI
**Weiss 2002 [**17**]**	Gastrointestinal surgery (23)	SO + FO^e^	SO	Mortality, Infection rate, H LOS, ICU LOS	IL-6^g^
**Wichmann 2007 [**18**]**	Major intestinal surgery (256)	SO/MCT/n-3 TGs^d^	SO	Mortality, Infection rate, H LOS, ICU LOS	AST, Bilirubin, TG, CRP, LTB5, LTB ratio, alpha-T, EPA, PT (Quick), Cr
Elective surgery, non-ICU patients (n = 740)					
**Badia-Tahull 2010 [**19**]**	Major gastrointestinal surgery (27)	SO + FO^e^	SO/OO)	Mortality, H LOS, Infection rate	ALT, CRP, TBU, Cr, PlU
**Grimm 2006 [**20**]**	Radical colorectal cancer resection (33)	SO/MCT/OO/FO ^b^	SO	H LOS^c^	alpha-T, AA, EPA, DHA, LTB4, LTB5, LTB ratio
**Jiang 2010 [**21**]**	Gastrointestinal malignancy (203)	SO + FO^e^	SO	H LOS, Infection rate, Bleeding events	IL-6, CrCl
**Klek 2005[**22**]**	Major abdominal surgery (58)	SO + FO^e^	SO/OO	H LOS, Infection rate	ALT, AST, Cr, PlU
**Koeller 2003 [**23**]**	Major abdominal surgery (30)	SO/MCT/n-3 TGs^d^	SO		LTB4, LTB5, LTB ratio
**Liang 2008 [**24**]**	Colorectal cancer (41)	SO + FO^e^	SO	Mortality, Infection rate, H LOS	IL-6
**Linseisen 2000 [**25**]**	Major abdominal surgery (33)	SO/MCT/n-3 TGs^cd^	SO		alpha-T, AA, EPA, DHA
**Makay 2011 [**26**]**	Major gastric surgery (26)	SO + FO^e^	SO	Mortality, Infection rate, H LOS	AST, ALT, Cr, PlU; Lac
**Mertes 2006 [**27**]**	Abdominal surgery (249)	SO/MCT/OO/FO^b ^	SO	Mortality, H LOS	AST, ALT, TG, Bilirubin
**Senkal 2007 [**28**]**	Major abdominal cancer (40)	SO/MCT/n-3 TGs^d^	SO/OO	Infection rate	AA, EPA, DHA

^a^Allocation to the ICU subgroup was driven by explicit mention in the published paper of an ICU stay, in the Methods or Results sections of the original article. ^b^SMOFlipid 20% (Fresenius Kabi): 1000 ml of emulsion contains: soya-bean oil, refined 60.0 g, triglycerides, medium-chain, 60.0 g, olive oil, refined 50.0 g, FO, rich in n-3-acids 30.0 g. ^c^Not used for meta-analysis as data are from a subgroup of Mertes *et al*.[27]. ^d^Lipoplus 20% (B.Braun): 1000 ml of emulsion contains: medium-chain triglycerides: 100.0 g, soybean oil, refined: 80.0 g, N-3-acid triglycerides: 20.0 g. ^e^Omegaven 10% fish oil emulsion (Fresenius Kabi): 100 ml emulsion contains highly refined fish oil 10.0 g containing eicosapentaenoic acid (EPA) 1.25 to 2.82 g, docosahexaenoic acid (DHA) 1.44 to 3.09 g, dl-α-tocopherol (as antioxidant) 0.015 to 0.0296 g. ^f^Data reported by graph only or qualitatively. ^g^No SD reported. AA, (%) content of arachidonic acid in serum/cellular membranes; alpha-T, alpha-tocopherol; ALT, alanine aminotransferase; AST, aspartate aminotransferase; BT, bleeding time; Cr, serum creatinine; CrCl, creatinine clearance; CRP, C-reactive protein; DHA,(%) docosahexaenoic acid content in serum/cellular membranes; EPA, (%) eicosapentaenoic acid content in serum/cellular membranes; FO, fish oil emulsion; Lac, lactate; (H) LOS, (Hospital) length of stay; ICU, intensive care unit; LTB, leukotriene B; LTB ratio, LTB5: LTB4; (n-3) TGs, (n-3) triglycerides; MCT, medium chain triglycerides; OI, oxygenation index; OO, olive oil emulsion; PlU, serum urea; PT, prothrombin time; PTT, partial thromboplastin time; SO, soybean oil emulsion; TBU, transfused blood units.

Checking for duplicate publication of the same data revealed that the studies by Antebi *et al*. [6] and Grimm *et al*. [20] were subgroup analyses of the same study fully published by Mertes *et al*. [27]. We therefore excluded from the analyses the values reported Antebi *et al*. [6] for aspartate aminotransferase (AST), alanine aminotransferase (ALT) and triglyceride levels, and the data on hospital LOS recorded in Grimm *et al*. [20], as all of these parameters were already in the Mertes *et al*. publication [27]. Hospital LOS data were reported as median and interquartile range by more than one study: the corresponding mean and SD values were estimated after fitting a Weibull distribution to reported data.

### Clinical Outcomes

The results of the pooled analyses with the random effects models (Table 3) indicate no statistically significant difference in mortality rate between patients receiving n-3 PUFA-enriched lipid emulsions and those receiving the standard lipid emulsions, soybean oil, MCT/LCT or olive/soybean oil emulsions. The time point at which mortality was assessed varied: while 28/30 days mortality was the most commonly used definition [7,9,16,18], in-hospital mortality was analysed in one report [19], and in the remaining studies [14,17,26,27] the endpoint for this outcome was not specified. This lack of clarity/homogeneity may somewhat hamper the interpretation of presented results.

**Table 3:** n-3 PUFA-enriched versus standard parenteral lipid emulsions for parenteral nutrition: random effects meta-analysis

Outcome	Studies	Patients (n)	Effect estimate
Mortality, overall RR	10	847	0.89 (0.59, 1.33)
ICU patients	7	547	0.94 (0.61, 1.45)
Non-ICU patients	3	300	0.58 (0.18, 1.84)
Infection rate, overall RR	11	919	0.61 (0.45, 0.84)*
ICU patients	5	524	0.71 (0.45, 1.12)
Non-ICU patients	6	395	0.53 (0.34, 0.82)*
Hospital LOS, overall MD	15	1169	-3.29 (-5.13, -1.45)*
ICU patients	8	615	-5.17 (-8.35, -1.99)*
Non-ICU patients	7	554	-1.86 (-3.13, -0.59)*
ICU LOS, MD	8	615	-1.92 (-3.27, -0.58)*
CRP, overall MD	7	432	-11.28 (-24.71, 2.16)
ICU patients	6	405	-9.76 (-23.57, 4.04)
Non-ICU patients	1	27	-46.00 (-108.12, 16.12)
IL-6 change, MD	3	105	37.70 (20.23, 55.16)*
Oxygenation index, MD	2	61	50.04 (10.99, 89.09)*
Serum lactate, MD	2	47	-0.29 (-1.38, 0.80)
LTB5, overall SMD	5	183	2.86 (1.22, 4.50)*
ICU patients	3	120	3.35 (0.54, 6.16)*
Non-ICU patients	2	63	2.14 (0.42, 3.85)*
LTB4, overall SMD	6	188	-0.47 (-1.18, 0.23)
ICU patients	4	125	-0.85 (-1.42, -0.27)*
Non-ICU patients	2	63	0.34 (-1.25, 1.92)
LTB ratio, overall MD	4	163	0.07 (0.05, 0.09)*
ICU patients	2	100	0.11 (0.01, 0.22)*
Non-ICU patients	2	63	0.06 (0.05, 0.07)*
EPA, overall SMD	9	271	4.12 (2.99, 5.25)*
ICU patients	6	165	4.65 (2.70, 6.60)*
Non-ICU patients	3	106	3.64 (2.65, 4.64)*
DHA, overall SMD	7	171	1.84 (0.65, 3.03)*
ICU patients	4	65	2.82 (0.17, 5.46)*
Non-ICU patients	3	106	1.33 (-0.11, 2.78)
Arachidonic acid, overall SMD	6	171	0.22 (-0.20, 0.64)
ICU patients	3	65	0.35 (-0.14, 0.84)
Non-ICU patients	3	106	0.14 (-0.62, 0.900
Alpha-tocopherol, overall MD	5	170	12.33 (8.73, 15.93)*
ICU patients	3	104	10.08 (5.39, 14.76)*
Non-ICU patients	2	66	15.25 (14.15, 16.35)*
Prothromin time (Quick), MD	2	300	0.43 (-2.62, 3.47)
Partial thromboplastin time, MD	2	65	10.71 (-30.08, 51.51)
Transfused blood units, SMD	2	209	-0.05 (-0.32, 0.22)
Platelet count	4	160	-6.32 (-31.40, 18.77)
Triglyceride level, overall MD	5	567	10.40 (-13.53, 34.34)
ICU patients	4	368	14.16 (-13.12, 41.44)
Non-ICU patients	1	199	-0.89 (-23.26, 21.48)
Serum creatinine, SMD	3	309	-3.01 (-7.11, 1.08)
Serum urea, SMD	2	53	-0.11 (-0.30, 0.08)
AST, overall MD	7	656	-10.05 (-18.81, -1.29)*
ICU patients	4	373	-10.11 (-27.31, 7.10)
Non-ICU patients	3	283	-8.37 (-17.36, 0.61)*
ALT, overall MD	7	482	-9.85 (-17.49, -2.21)*
ICU patients	3	109	-18.18 (-21.68, -14.68)*
Non-ICU patients	4	373	-4.97 (-9.62, -0.32)*
Serum bilirubin, overall MD	4	520	0.03 (-0.33, 0.40)
ICU patients	3	321	0.12 (-0.42, 0.65)
Non-ICU patients	1	199	-0.02 (-0.17, 0.13)
			

Values in parentheses represent 95% CI. *Significant effect (*P *< 0.05). ALT, alanine aminotransferase; AST, aspartate aminotransferase; CRP, C-reactive protein; DHA,(%) docosahexaenoic acid content in serum/cellular membranes; EPA, (%) eicosapentaenoic acid content in serum/cellular membranes; (H) LOS, (Hospital) length of stay; ICU, intensive care unit; LTB, leukotrienes B ; LTB ratio, LTB5: LTB4; MD, mean difference; RR, relative risk; SMD, standardised mean difference.

**Figure 1:**
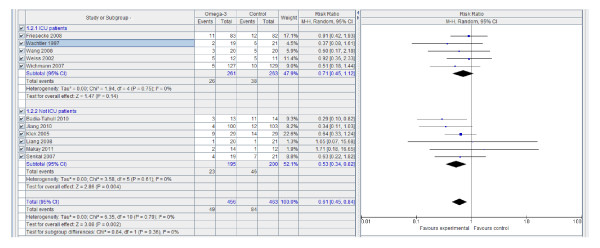
**Infection rate: random effects meta-analysis and forest plot**. Squares represent individual study mean of the effect measure, diamonds represent its pooled estimates.

**Figure 2:**
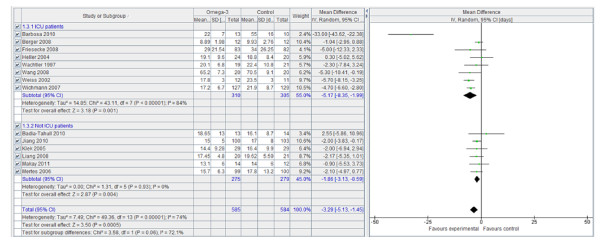
**Hospital length of stay: random effects meta-analysis and forest plot**. Squares represent individual study mean of the effect measure, diamonds represent its pooled estimates.

**Figure 3:**
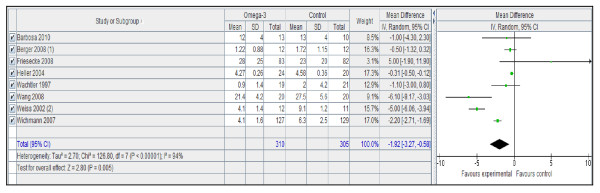
**ICU length of stay: random effects meta-analysis and forest plot**. Squares represent individual study mean of the effect measure, diamonds represent its pooled estimates.

However, n-3 PUFA-enriched regimens are associated with a statistically and clinically significant reduction in the infection rate (Figure 1) and LOS, both in the ICU (Figure 2) and in hospital overall (Figure 3). If only ICU patients' data are considered, the reduction in infection rate is not statistically significant.

Lung gas exchange (oxygenation index), measured in only two studies in septic ICU patients [7,16], was significantly increased in patients receiving 3 PUFA-enriched parenteral nutrition regimens. No statistically significant differences between treatments could be detected for bleeding-related outcomes, including blood transfusion requirements.

### Markers of inflammation, antioxidant status and fatty acid composition of plasma phospholipids

**Figure 4:**
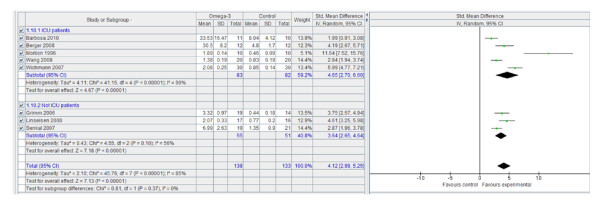
**EPA concentration in plasma phospholipids: random effects meta-analysis and forest plot**. EPA: eicosapentaenoic acid. Squares represent individual study mean of the effect measure, diamonds represent its pooled estimates.

**Figure 5:**
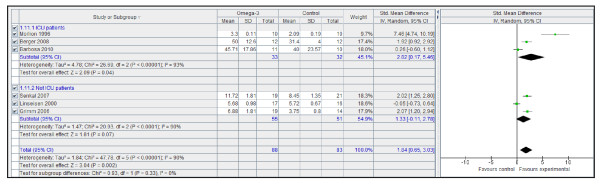
**DHA concentration in plasma phospholipids: random effects meta-analysis and forest plot**. DHA: docosahexaenoic acid. Squares represent individual study mean of the effect measure, diamonds represent its pooled estimates.

Use of n-3 PUFA-enriched emulsions significantly increases the serum concentration of alpha-tocopherol and the percentage content of EPA (Figure 4) and DHA (Figure 5) in phospholipids. However, the content of AA in phospholipids was unchanged by n-3 PUFA enrichment.

**Figure 6:**
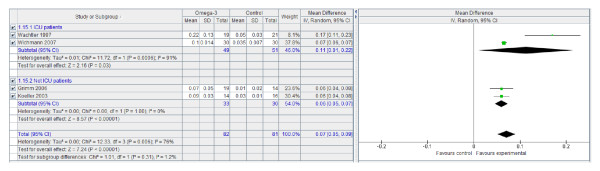
**LTB5/LTB4 production: random effects meta-analysis and forest plot**. LT: leukotriene. Squares represent individual study mean of the effect measure, diamonds represent its pooled estimates.

There was a significantly greater reduction in IL-6 and a shift in the generation of leukotrienes towards the leukotriene-5 series, as indicated by the significant absolute increase in leukotriene B5 (LTB5), the absolute decrease of LTB4, and the significantly ameliorated LTB5: LBT4 ratio (Figure 6).

### Routine laboratory parameters

The analysis indicates a significant reduction in serum ALT and AST, with n-3 PUFA-enriched emulsions in comparison to standard lipid emulsions. However, no significant differences between the interventions were detected in coagulation times, platelet count, serum levels of triglycerides, C-reactive protein (CRP), or bilirubin.

### Fixed effect models

In addition to the differences detected by the random effects models, fixed effect models also indicate a significant effect of n-3 PUFA-enrichment regimens on AST levels in ICU patients; bilirubin, CRP, and triglycerides in the overall and ICU populations; docosahexaenoic acid (DHA) in non-ICU patients, and LTB4 in the overall population (data not shown).

## Discussion

A total of 23 studies (n = 1,502 patients; n = 762 admitted to the ICU) were included in our meta-analysis. Pooled data indicate important and significant positive effects of n-3 PUFA-enriched parenteral regimens over a wide range of outcomes in the selected patient populations. Subgroup analysis shows that the magnitude of these effects for some of the outcomes varied between ICU patients and elective surgery patients. In some cases, although the effect is estimated as statistically significant for the whole considered population, this is not true for one or both subgroups, very probably as a consequence of the reduced patient number in the analysed population. Some differences in laboratory parameters (bilirubin, triglycerides, CRP, LTB4; data not shown) were not statistically significant with the random effects model, but were statistically significant when analysed with the less conservative, fixed effects model. For five pre-specified outcomes, the incidence of systemic inflammatory response syndrome (SIRS), bleeding events, international normalized ratio (INR), bleeding time, and creatinine clearance, data were qualitatively or quantitatively insufficient to obtain a meta-analytic effect estimate.

There are no clear data from RCTs on optimum doses, while a case series analysis indicates that the optimal dose may be related to the diagnosis [29]. Dosages used are mainly related to body weight, and if anything can be observed from existing data, the dose at least, must exceed 0.1 to 0.15 g/Kg/d fish oil. In order to address this question, a clinical trial for optimal dose determination has been designed and is being conducted (FOILED study: ClinicalTrials.gov NCT01146821). Regarding the optimal moment for starting parenteral lipids (pre- and/or post-event), and possible effects on outcome, there are no inferential data, although logical thinking may suggest the earliest possible intervention.

The available pooled evidence in the present study on 3 PUFA-enriched parenteral regimens, in particular EPA and DHA, in surgical and ICU patients presenting with inflammatory conditions, indicates that their use is safe and effective in reducing the morbidity burden and the required hospitalization period. Moreover, results suggest that the hypothesised mechanism by which these effects are attained is plausible.

With n-3 PUFA-enrichment, the content in phospholipids clearly shifts its balance towards the n-3 series, as indicated by relevant and statistically significant increases in EPA and DHA levels, without a significant decrease in AA, which is reassuring in light of the important physiological functions modulated by the latter. The more favourable balance of n-3 versus n-6 PUFAs is reflected in the increased release of less pro-inflammatory leukotrienes, such as LTB5, and particularly in the ratio between these and more pro-inflammatory cell messengers (LTB5/LTB4), and also by the reduction in cytokines, such as IL-6, and of the inflammation marker CRP (significantly reduced according to the fixed effects model, but with a non-significant trend towards reduced levels also in the more conservative random effects model). Actually, regarding the velocity of IL-6 reduction after peaking, there is more evidence in favor of n-3 PUFAs-enriched regimens than was possible to include in the quantitative data pooling: Jiang *et al*. [21], Wachtler *et al*. [15] and Weiss *et al*. [27] all report that IL-6 was increased in controls, and reduced in patients receiving n-3 PUFA-enriched regimens, and Wang *et al*. [16] report a significant difference in favour of n-3 PUFA-enriched regimens, but none of these reports included adequate quantitative data.

Lung gas exchange assessed by the oxygenation index is better-preserved or improved in patients receiving n-3-enriched lipid emulsions than with standard lipid emulsions. As for safety of use, no significant differences among treatment groups could be detected in terms of coagulation parameters, either for laboratory markers, or the clinical outcome of blood transfusion requirements. The same holds true for renal function: serum creatinine and urea were not significantly different among treatment groups, and if anything can be deduced from the data, this is a trend towards less impairment of kidney function with n-3 PUFA-enriched emulsions. Liver enzymes are significantly less increased in patients receiving n-3 PUFA-enriched emulsions than in those treated with standard lipid emulsions, suggesting a possible hepatoprotective action of fish oil components, which should be studied further.

The clinical results obtained are consistent with laboratory findings: although mortality is not significantly affected, there is a clear advantage in terms of infective complications and a relevant improvement in recovery times, as indicated by the significant reductions in the ICU and hospital LOS. There is a not a significant trend towards decreased mortality: possible explanations of the failure to show a significant effect include the low mortality risk in elective surgery patients (< 5% in the considered control groups) and the low overall patient numbers for high-mortality studies. However, the absence of a mortality difference increases the value of the LOS reduction, as it cannot be argued that this effect is a consequence of increased mortality.

The fact that these results were obtained in studies in which different formulations of n-3 PUFAs were compared with a range of alternative lipid emulsions further strengthens the concept of using lipid emulsions that include the n-3 PUFA EPA and DHA. Our results confirm and extend the scope of those obtained in the earlier analysis in surgical patients by Chen *et al*. [5]. We included a greater number of studies and also evaluated data collected from patients admitted to ICU. The only relevant difference between outcomes considered in both analyses is the reduced leukocyte LTB4 production, which reached statistical significance with the fixed effects model.

## Conclusions

In conclusion, these results confirm previous findings in surgical patients and extend them to the ICU population: the body of available evidence indicates that the use of n-3 PUFA-enriched parenteral nutrition is safe and effective in reducing the infection rate and hospital/ICU stay in surgical patients, and that these benefits also apply to ICU patients. Other beneficial effects included reduced markers of inflammation, improved lung gas exchange, liver function, antioxidant status and fatty acid composition of plasma phospholipids, and a trend towards less impairment of kidney function.

## Key messages

• Enriching conventional lipid emulsions with n-3 PUFA results in a statistically and clinically significant reduction in the infection rate and the length of stay in the ICU, and in postsurgical patients receiving parenteral nutrition.

• Mortality is not decreased, possibly because of the low mortality risk in the patient group as a whole.

• n-3 PUFA-enriched parenteral nutrition regimens are well-tolerated and there is a trend towards less impairment of kidney function, as well as significantly improved liver function and lung gas exchange.

## Abbreviations

AA: arachidonic acid; ALT: alanine aminotransferase; AST: aspartate aminotransferase; CRP: C-reactive protein; DHA: docosahexaenoic acid; EPA: eicosapentaenoic acid; ICU; intensive care unit; LCT: long-chain triglyceride; LTB: leukotriene B; LOS: length of stay; MCT: medium-chain triglyceride; OI: oxygenation index; PT: prothrombin time; PPT: partial thromboplastin time; PUFA: polyunsaturated fatty acids; RCT: randomized clinical trial; RR: relative risk; SMD: standardised mean difference.

## Competing interests

Dr. Lorenzo Pradelli is co-owner and employee of AdRes, which has received project funding by Fresenius Kabi. Prof. Axel Heller received speaker honoraria and project funding by BBraun, Melsungen, Germany and by Fresenius- Kabi, Bad Homburg, Germany. Prof. Maurizio Muscaritoli received speaker honoraria by Baxter, BBraun, and Fresenius Kabi. K. Mayer received fees for product neutral lectures and compensation for travel costs from Abbott, Baxter, BBraun, Fresenius Kabi, Nestle, Pfizer.

## Authors' contributions

LP conceived the study, extracted clinical data, performed the statistical analysis and drafted the manuscript. KM participated in the design of the study, reviewed the literature and helped to draft the manuscript. MM participated in the design of the study, reviewed the literature and helped to draft the manuscript. AH participated in the design of the study, reviewed the literature and helped to draft the manuscript. All authors read and approved the final manuscript.

## Supplementary Material

Additional file 1 **Figure S1. Prisma flowchart of study selection**. Click here for file
