# Supplementary material for: n-3 fatty acid-enriched parenteral nutrition regimens in elective surgical and ICU patients: a meta-analysis
Source: Crit Care. 2012 Oct 4;16(5):R184. doi: 10.1186/cc11668 (PMC3682286; doi:10.1186/cc11668)
Supplement: Additional file 1 — Figure S1. Prisma flowchart of study selection. [file cc11668-S1.DOC]

**Screening**

**Included**

**Eligibility**

**Identification**

Records identified through database searching
(n = 52)

Additional records identified through reference check
(n = 8)

Records after duplicates removed
(n = 60)

Records screened
(n = 60)

Records excluded
(n = 30 )

Full-text articles assessed for eligibility
(n = 30 )

Full-text articles excluded, with reasons
(n =7, 3 no outcomes, 4 wrong populalation)

Studies included in qualitative synthesis
(n = 23)

Studies included in quantitative synthesis (meta-analysis)
(n = *)
